# Ground nesting by arboreal American robins (*Turdus migratorius*)

**DOI:** 10.1002/ece3.8489

**Published:** 2022-01-17

**Authors:** Sarah K. Winnicki, Mark E. Hauber, Thomas J. Benson, Mikus Abolins‐Abols

**Affiliations:** ^1^ Department of Evolution, Ecology, and Behavior School of Integrative Biology University of Illinois at Urbana‐Champaign Urbana Illinois USA; ^2^ Program in Ecology, Evolution, and Conservation University of Illinois at Urbana‐Champaign Urbana Illinois USA; ^3^ Illinois Natural History Survey Prairie Research Institute University of Illinois at Urbana‐Champaign Champaign Illinois USA; ^4^ Department of Biology University of Louisville Louisville Kentucky USA

**Keywords:** microclimate, nest placement, predation, temperature

## Abstract

Animals with dependent and vulnerable young need to decide where to raise their offspring to minimize ill effects of weather, competition, parasitism, and predation. These decisions have critical fitness consequences through impacting the survival of both adults and progeny. Birds routinely place their nest in specific sites, allowing species to be broadly classified based on nest location (e.g., ground‐ or tree‐nesting). However, from 2018 to 2020, we observed 24 American robin (*Turdus migratorius*) nests placed not on their species‐typical arboreal substrates or human‐made structures but on the ground at a predator‐rich commercial tree‐farm in Illinois, USA. This behavior does not appear to be in response to competition and did not affect nest daily survival rate but was restricted to the early half of the breeding season. We hypothesize that ground nesting may be an adaptive response to avoid exposure and colder temperatures at sites above the ground early in the breeding season or a nonadaptive consequence of latent robin nest‐placement flexibility.

## Graphical abstract

Mobile organisms often construct breeding structures in set locations, but American Robins that typically nest in trees or on human structures construct ground nests in predator‐rich tree‐filled arbors in Central Illinois, USA. Robins build these ground nests in the early breeding season, when tree nests are often quite visible to the human eye.

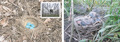

## 1 INTRODUCTION

Locating a site suitable for successful reproduction is an essential task of many mobile organisms. Breeding animals often need to find denning, burrowing, or other nesting locations that simultaneously provide access to nearby resources and limit resource competition while, critically, also protect both the parents and developing offspring from adverse weather/microclimates, parasites, competitors, and/or predators (Mainwaring et al., 2016). These constraints have led to many species evolving to use relatively consistent breeding sites and substrates among individuals. Accordingly, most songbird species can be readily classified into categories such as tree, ground, or cavity nesters (Nagy et al., 2019), although some species show plasticity in nest site preference depending on the shifting habitat structure (e.g., song sparrows (*Melospiza melodia*) raise their nest height from the ground to shrubs as the breeding season progresses: Morse Nice, 1931). Other species may shift their nest placement to avoid predators (e.g., orange‐crowned warblers (*Vermivora celata*) place nests increasingly off the ground and into shrubs on islands where ground predators are more prevalent and avian predator pressure is reduced: Peluc et al., 2008). Observing such instances of nest‐site switching allows us to address both the drivers of this behavioral plasticity and their implications for reproductive success.

American robins (*Turdus migratorius*, hereafter “robin”) are an iconic backyard songbird species in North America that build a bulky mud‐lined nest off the ground in trees, shrubs, and human‐made structures (Vanderhoff et al., 2020). Robins are classified as arboreal nesters (Vanderhoff et al., 2020); there are only two published examples of ground‐nesting robins and these are both from “extreme” environments among what is representative for this species, for example, where trees and shrubs are altogether absent (e.g., in tilled soyfields in Central Illinois, VanBeek et al., 2014) or on islands with no mammalian predators (Collias, 1964). However, we observed robins nesting on the ground at a tree‐dense *and* predator‐rich study site over multiple study years.

## 2 METHODS AND RESULTS

As part of our ongoing studies of robins and their anti‐parasitic egg rejection behaviors (e.g., Abolins‐Abols & Hauber, 2020; Hauber, 2020), from 2018 to 2020, we located 1,170 robin nests at a commercial deciduous tree farm from April to July in Champaign County, Illinois, USA, including 24 robin nests built directly on the ground. We found nests by searching each tree row and the surrounding ground every 3 days throughout the season, visually locating the bulky nests and following vocalizing or flushed adults. Once the growing tree leaves obscure nests later in the season, we extended our searching effort to thoroughly search the trees while continuing to search the ground later in the year. Notably, we only found ground nests early in the breeding season (initiated between 22 April and 12 May) while we found tree nests throughout the spring and summer (initiated between 8 April and 30 June). Whereas most of the nests were built in trees and shrubs and the ground nests represented a minority (2%) of nests we found, it is noteworthy that ground nests were present in all 3 years and that multiple ground nests were active simultaneously, indicating that this ground‐nesting behavior was not limited to a single individual female robin within and across years.

**FIGURE 1:**
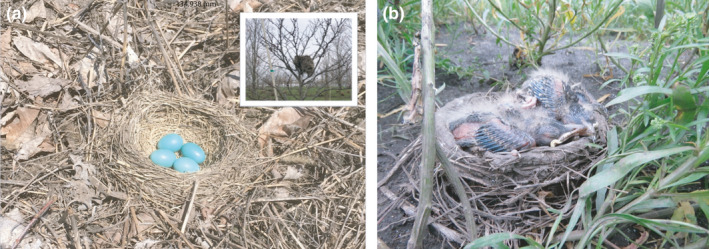
(a) An American robin (*Turdus migratorius*) ground‐nest in the incubation stage. To human observers, the nest is well‐camouflaged against a litter substrate but the bright blue eggs are readily visible when the incubating female is not present. This stands in contrast to typical tree nests early in the season, which are readily visible to human observers (inset). Photo credits: M. Hauber. (b) A robin ground‐nest in the nestling stage. Four nestlings are present in the nest cup, which sits in a small depression in the ground. The rim of the cup is built‐up mud and grass like an arboreal robin nest and extends <5 cm above the ground. Photo credit: S. Winnicki

The ground nests were located near deciduous tree saplings or at the bottom of ~0.5 m‐deep holes in the ground left by commercial tree‐and‐root removal at our commercially active site. The substrates around the nests included bare dirt and mud, thick dead plant litter, or growing grasses and forbs. The nests consisted of a depression in the ground and a low (<5 cm tall) circular rim of dried mud and plant material above the ground and were lined with fine plant materials, similar to arboreal robin nests (Figure 1). The placement of the nests, integrated into depressions on the ground, suggests that the nests were constructed there, rather than moved to the ground during the tree‐removal process at this commercial site.

The clutches in the ground nests contained 2–4 eggs (mean ± SD: 3.00 ± 0.71), comparable to tree‐nesting robin nests (3–4; Vanderhoff et al., 2020), and our local data (range: 1–5, mean ± SD: 2.78 ± 1.02). Predators are abundant in this region of Illinois, depredating as much as 45% of monitored songbird nests (Chiavacci et al., 2018), and abundant at the site; nest cameras at local arbors show that raccoons (*Procyon lotor*), snakes (fox snakes [*Pantherophis ramspotti*], and garter snakes [*Thamnophis sirtalis*]), and avian predators (Brown‐headed cowbirds [*Molothrus ater*], Cooper's hawks [*Accipiter cooperii*], and common grackles [*Quiscalus quiscula*]) regularly depredate tree nests (our unpublished data). Most of the tree nests we located at the sites were manipulated for other experiments (e.g., Abolins‐Abols & Hauber, 2020; Hauber, 2020) and this limited our nest success analysis of the tree‐nest data to the 185 nests that we monitored but did not manipulate in 2019. Of the 19 ground nests with known outcome from all years, at least eight (44%) survived until the eggs hatched but only two (11%) successfully produced fledglings compared to 25% of the 2019 tree nests that made it to hatch and 17% survived to produce fledglings. Daily nest survival rates (calculated using *RMark* [Laake, 2013]) were 0.891 (95% confidence interval (CI_95%_): 0.869–0.909) for tree nests across the entire breeding season in 2019 (the year for which we have the most such data from experimentally unmanipulated tree nests) and 0.868 (CI_95%_: 0.795–0.917) for ground nest across all 3 years. Tree nests that were active at the same time as ground nests (initiated prior to 12 May, which was the latest initiation date for ground nests) had a daily survival rate of 0.877 (CI_95%_: 0.840–0.907), while tree nests initiated after 12 May had a daily survival rate of 0.900 (CI_95%_: 0.873–0.922).

## 3 DISCUSSION

A subset of the robins at our tree‐dominated study site exhibit nest‐placement flexibility, building their nests on the ground rather than in shrubs or on trees in the early half of the breeding season. The decision to use a ground‐level nest site could be the result of competition for suitable tree sites. Limited sites are a known driver of nest placement and a constraint on the number of breeding individuals, although this is a pattern most often seen in cavity‐nesting birds rather than open‐cup nesting birds (Newton, 1994). Contrary to this hypothesis, most of the robin ground nests we observed were located directly under or within 5 m of seemingly suitable unoccupied trees, suggesting that competition for nesting trees was not the primary driver of the robins' decision to build their nest on the ground at this site.

Because predation is the primary cause of nest failure in songbirds (Martin, 1995), avoiding predators can also drive nest‐placement decisions. Nesting birds may face a tradeoff between choosing sites with enough cover to conceal their nest and enough visibility for incubating and brooding parents to see potential threats (Götmark et al., 1995). Ground nests could therefore be beneficial because of the increased visibility they afford to robin parents, while simultaneously providing more concealment from potential predators. Predator identity may also matter; while nesting on the ground may reduce the likelihood of depredation by avian predators and arboreal mammals, nesting in trees may make nests less accessible to mostly terrestrial predators like raccoons, rat snakes, and deer observed at nearby songbird nest monitoring sites (Chiavacci et al., 2018), and nests placed higher are often safer overall (Burhans et al., 2002). While our small sample size for ground nests prevented us from statistically comparing daily survival rates between ground and tree nests, the survival rates are qualitatively similar, suggesting that flexibility in nest placement does not dramatically impact predation‐driven nest failure overall.

**FIGURE 2:**
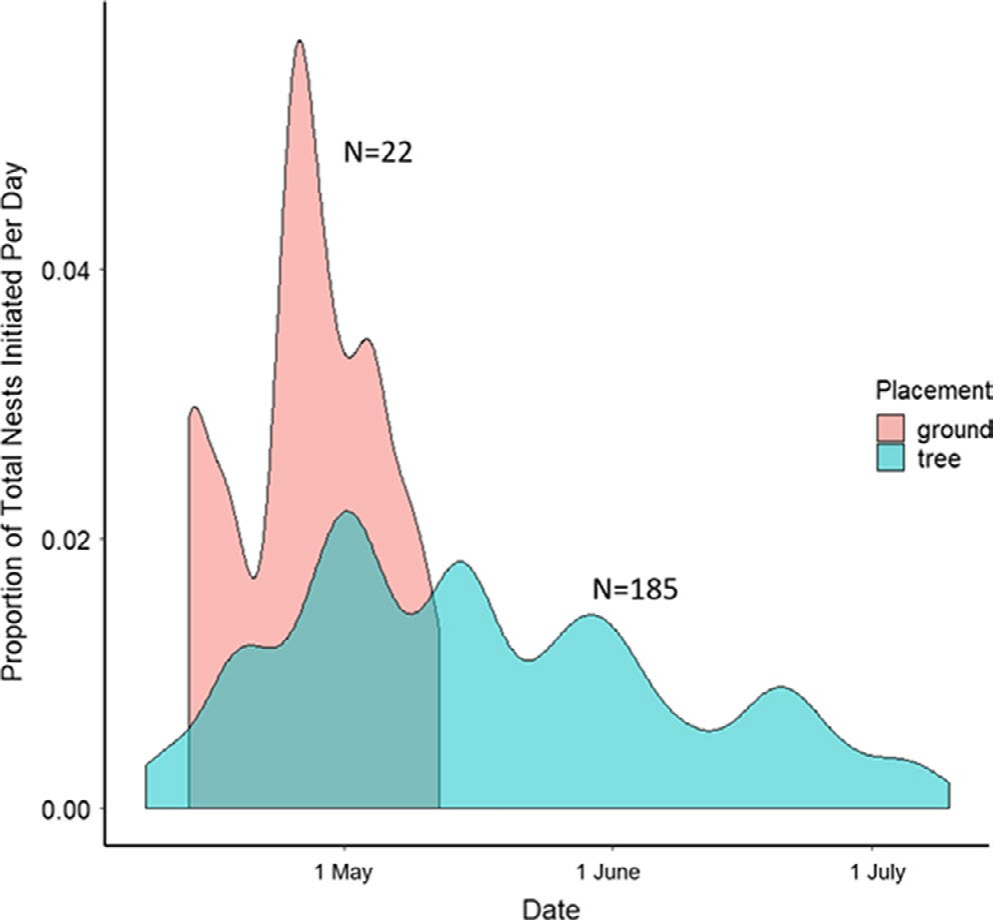
The 22 ground nests (red‐shaded curve) for which we could estimate laying date were initiated early in the breeding season, in April and May. This contrasts with the 185 tree nests from 2019 (blue‐shaded curve), which were initiated from April and May into June and July

Whereas daily survival rates suggest that ground‐nesting robins do not suffer increased predation risk overall, it is possible that the relative predation risk for ground‐ and tree‐nesting birds varies across the season. Many robins at our site initiate nesting before the planted trees have leafed out, making the bulky robin nest structures built in barren trees clearly visible to human observers (Figure 1 inset), which might cause some robin females to show a seasonal preference for building nests on the ground. This seasonal leaf‐limitation hypothesis predicts that ground nesting should be beneficial relative to tree‐nesting in the beginning of the breeding season when the leaves are not present. Accordingly, all of our ground nests had been initiated in April and early May, when tree leaves are absent or still growing; we found no ground nests initiated after 12 May despite our nest searching extending until July 4 each year (Figure 2). While it is possible that nest detectability changes throughout the season, we increased our nest‐searching effort to find obscured tree nests in leafed‐out trees and continued to search the ground systematically. The forbs and grasses in‐between the tree rows grow during the season, potentially obscuring ground nests, but many of the ground nests found earlier in the season were located on the bare ground at the base of the trees; we found no ground nests in these visible locations after 12 May. In contrast to the ground nests, we regularly located many arboreal nests initiated from April through early July (Figure 2). Although we could not statistically compare the daily survival rates of the ground nests and tree nests initiated during the same time period (the early half of the season), the rates were again qualitatively similar, suggesting that ground nests were not more likely to survive than the exposed tree nests.

An alternative hypothesis is that nest placement may be an important determinant of the immediate weather and microclimate conditions experienced by the incubating/brooding parents and the developing offspring (Ardia et al., 2006). Cup nests in trees in the open air experience a different microclimate than nests partially in the ground. The early half of the breeding season, when we found all of our ground nests, is considerably colder (average for Champaign County in April and May: low of 5°C, high of 23°C) than the second half of the breeding season (average for June and July: low of 17°C, high of 29°C, State Climatologist Office for Illinois). It is possible that nests in depressions on the ground retain heat better than nests in trees, benefiting ground‐nesters over tree‐nesters when the temperatures are cold, a prediction that could be readily tested with temperature monitors in nests throughout the season. These conditions may present a trade‐off for the birds between choosing sites with a more suitable microclimate or lower predation risk, as is the case in some desert species (Tieleman et al., 2008), although the equivalent survival rates between tree and ground nests in our study site argue against this trade‐off.

Even though it is not immediately clear why some of the otherwise overwhelmingly arboreal robins in our system are nesting on the ground within a tree farm, such behavioral plasticity in life history traits could be consequential for the robins' ability to adjust to changing environments, including ongoing anthropogenic and climate change (Mainwaring et al., 2016). For example, dark‐eyed juncos (*Junco hyemalis*) colonizing novel, urban habitats have adjusted both their nest placement and nest reuse patterns, producing more successful offspring compared to forest dwelling populations (Yeh et al., 2007). Although our study sites are inside a strictly agricultural matrix, they are also within 10 km of a dense and urban robin population in Urbana‐Champaign, suggesting that more flexibly nesting urban robins may be the source of variation in nest site choice in our study. Future work should elucidate not only the environmental correlates of the ground‐nesting behavior but also the fitness consequences of nest placement in this still common species. Indeed, ground nesting at our site may not be an adaptive response to local environmental conditions, but, for example, due to immigration of robins with preference for ground nesting due to previous experience, due to the breeding adult age or inexperience, and/or vertical transmission of nest‐site preference from ground‐nesting parents (Slagsvold et al., 2013).

## CONFLICT OF INTEREST

The authors declare no conflicts of interest.

## AUTHOR CONTRIBUTIONS

**Sarah K. Winnicki:** Conceptualization (equal); data curation (equal); formal analysis (lead); investigation (equal); methodology (equal); visualization (lead); writing – original draft (lead); writing – review and editing (lead). **Mark E. Hauber:** Conceptualization (equal); data curation (supporting); funding acquisition (lead); investigation (equal); methodology (equal); project administration (equal); supervision (lead); visualization (supporting); writing – original draft (supporting); writing – review and editing (equal). **Thomas J. Benson:** Conceptualization (equal); investigation (equal); methodology (equal); supervision (equal); writing – review and editing (equal). **Mikus Abolins‐Abols:** Conceptualization (equal); data curation (equal); formal analysis (supporting); investigation (equal); methodology (supporting); supervision (equal); writing – review and editing (supporting).

## Data Availability

Tree nest data are publicly available at https://doi.org/10.6084/m9.figshare.14195141. Ground nest data are publicly available at https://doi.org/10.6084/m9.figshare.14195123.
